# Synthetic Scaffold Systems for Increasing the Efficiency of Metabolic Pathways in Microorganisms

**DOI:** 10.3390/biology10030216

**Published:** 2021-03-11

**Authors:** Almando Geraldi, Fatiha Khairunnisa, Nadya Farah, Le Minh Bui, Ziaur Rahman

**Affiliations:** 1 Department of Biology, Faculty of Science and Technology, Universitas Airlangga, Surabaya 60115, Indonesia; 2 Research Center for Bio-Molecule Engineering, Universitas Airlangga, Surabaya 60115, Indonesia; fatiha.khairunnisa@fst.unair.ac.id; 3 Department of Chemistry, Faculty of Science and Technology, Universitas Airlangga, Surabaya 60115, Indonesia; 4 Department of Biology, Faculty of Mathematics and Life Sciences, Indonesia Defense University, Bogor 16810, Indonesia; nadya.farah@idu.ac.id; 5 NTT Hi-Tech Institute, Nguyen Tat Thanh University (NTTU), Ho Chi Minh City 700000, Vietnam; blminh@ntt.edu.vn; 6 Department of Microbiology, Abdul Wali Khan University Mardan, Mardan, Khyber Pakhtunkhwa 23200, Pakistan; ziamicrobiologist@gmail.com

**Keywords:** metabolic engineering, synthetic biology, protein scaffolds, RNA scaffolds, DNA scaffolds

## Abstract

**Simple Summary:**

Biotechnology involves the use of living organisms to create high-value products. Bacteria and yeast, in particular, are widely applied for such processes, but they may not naturally produce certain products, such as amino acids, organic acids, and alcohols in large amounts, if at all. Hence, the field of metabolic engineering has emerged for “tweaking” the biosynthetic pathways of these cells to encourage the high production of desired products. However, the complexity of the many metabolic pathways in natural cells makes it difficult to ensure that only the molecular components and pathways related to the desired product are enhanced. Very often, competing metabolic pathways and toxic intermediates will lower the production efficiency. Biological scaffolds have thus emerged as one strategy for anchoring the correct enzymes and substrates in place, and in the right orientation, to improve the production process in the cells. This review introduces the different categories of molecular scaffolds (i.e., protein, RNA, and DNA scaffolds) that have been developed, and compares their pros and cons and enhancement of production yields. It is emphasized that the design of these scaffolds is still a trial-and-error process, and further studies are needed to shed light on their underlying mechanisms so that better scaffolds can be developed.

**Abstract:**

Microbes have been the preferred hosts for producing high-value chemicals from cheap raw materials. However, metabolic flux imbalance, the presence of competing pathways, and toxic intermediates often lead to low production efficiency. The spatial organization of the substrates, intermediates, and enzymes is critical to ensuring efficient metabolic activity by microorganisms. One of the most common approaches for bringing the key components of biosynthetic pathways together is through molecular scaffolds, which involves the clustering of pathway enzymes on engineered molecules via different interacting mechanisms. In particular, synthetic scaffold systems have been applied to improve the efficiency of various heterologous and synthetic pathways in *Escherichia coli* and *Saccharomyces cerevisiae*, with varying degrees of success. Herein, we review the recent developments and applications of protein-based and nucleic acid-based scaffold systems and discuss current challenges and future directions in the use of such approaches.

## 1. Introduction

**Figure 1:**
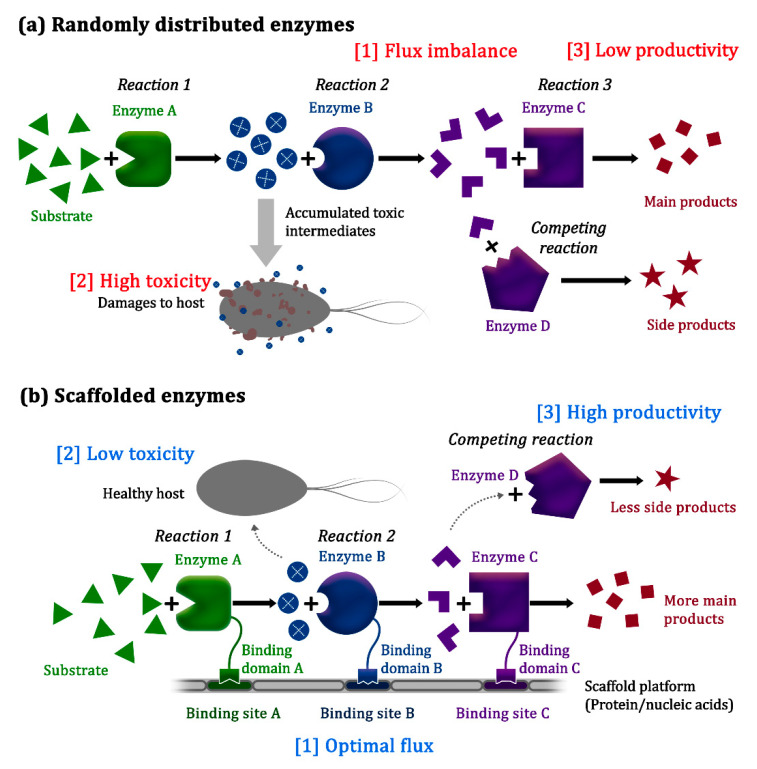
Spatial organization of pathway enzymes for efficient biosynthetic reactions. (**a**) Without spatial organization, pathway enzymes are randomly distributed, and their substrates also diffuse freely in the cell, which might result in (1) flux imbalance due to varying enzyme kinetics, (2) reduced cell viability due to the accumulation of toxic intermediates, and (3) low productivity due to diversion of the metabolic flux through competing pathways. (**b**) Synthetic scaffold systems organize the pathway enzymes and facilitate the substrate channeling effect for increasing pathway efficiency, thereby limiting the accumulation of toxic intermediates and reducing flux diversion to competing pathways [10].

Metabolic engineering involves the use of rational approaches for altering the metabolic pathways of an organism [1,2], with the goal being to enable the economical and sustainable production of valuable chemicals that are currently being derived from non-renewable or limited natural resources by using simple, readily available, and inexpensive starting materials instead [3,4,5]. Since its emergence in the 1990s, metabolic engineering has been successfully utilized for the production of active pharmaceuticals, biopolymers, and biofuels, mainly in microorganisms [6,7]. However, efforts to engineer various biosynthetic pathways, such as by overexpressing heterologous enzymes, and modulating the expression levels of metabolic pathway enzymes, have often been hampered by low production yields caused by imbalance of the pathway flux, undesirable crosstalk with other cellular pathways, and the compromised viability of host cells due to the accumulation of toxic intermediates (Figure 1a) [8,9].

In natural systems, balancing the metabolic flux to create the ideal metabolic pathway is achieved through spatial organization of the reactants, intermediates, and enzymes involved in the pathway [11,12,13]. In eukaryotic cells, components of diverse metabolic pathways are sequestered in organelles, such as mitochondria, chloroplast, and vacuoles [14,15]. Likewise, some metabolic pathways in prokaryotes are localized in protein-based bacterial microcompartments (BMCs) [16]. Carboxysomes are the BMCs involved in CO_2_ fixation, while metabolosomes sequester enzymes involved in and intermediates resulted during the oxidation of alternative carbon sources (such as propanediol, ethanolamine and choline) [17,18,19]. Spatial organization of a metabolic pathway can also be achieved through the arrangement of multi-enzyme complexes. The enzymes involved in the degradation of cellulose in the cellulosome of *Clostridium thermocellum* are co-localized with the cellulose-binding modules, which promote the accessibility and efficient degradation of the substrates [20,21]. Another example is the localization of multiple catalytic domains in complex enzymes, such as polyketide synthase, fatty acid synthase, and non-ribosomal peptide synthase [10,22].

Inspired by natural systems, researchers have developed synthetic platforms to spatially organize the enzymes involved in a specific metabolic pathway (Figure 1b). Macromolecules such as proteins, RNA, and DNA have been used as frameworks for confining the enzymes of interest to facilitate efficient transfer of the intermediates, thereby preventing the activity of other competing reactions and the accumulation of toxic intermediates [23,24].

To date, protein domains, synthetic RNA structures, and DNA molecules have been engineered as scaffolds and applied to various pathways in bacteria and yeast, with varying degrees of success. In this review, we cover recent applications of synthetic scaffold systems for enhancing metabolic pathway efficiency, and discuss the challenges and future directions of their use in the various metabolic engineering fields.

## 2. Protein Scaffolds

The use of peptide linkers for fusing two (or more) enzymes together represents the early efforts in organizing metabolic pathway enzymes in a spatial and temporal manner. This strategy could promote direct substrate channeling between enzyme active sites, as demonstrated by the successful enhancements of the muconic acid, α-farnesene and *n*-alkanes yields in *Escherichia coli* [25,26,27], and the resveratrol yield in *Saccharomyces cerevisiae* [28]. However, the application was limited to the ability of the host cells to efficiently express the folded and active enzyme fusion correctly [24,29]. Furthermore, the ratio of enzymes in the fusion was fixed to 1:1, which might not be optimal for most metabolic pathways [8].

The interactions between protein domains were manipulated to co-localize multiple metabolic enzymes on a scaffold system (Table 1). Inspired by the function of the cellulosome, researchers developed a trifunctional scaffold on the basis of the high-affinity interactions between three cohesion–dockerin domain pairs for co-localization of the NAD^+^-dependent dehydrogenase enzymes involved in methanol oxidation on the yeast cell surface [30]. The substrate channeling effect of the scaffolds resulted in a 5-fold increase in the nicotinamide adenine dinucleotide (NADH) production rate. Moreover, the co-localization of acetolactate synthase, acetolactate decarboxylase, and 2,3-butanediol dehydrogenase in *S. cerevisiae* improved the rate of pyruvate conversion to 2,3-butanediol by 1.4-fold [31].

The abundance of interacting protein domains gave rise to various protein scaffolding strategies. In one study, leucine zipper proteins were used to co-localize the enzymes involved in the butanol production pathway in cellulose-binding domains, which successfully increased the 1-butanol yield by 1.5-fold [41]. In another study, the *E. coli* inner cell membrane was used as the enzyme-docking platform to improve the efficiency of metabolic pathways involving membrane-bound substrates, such as β-carotene conversion to astaxanthin. The co-localization of β-carotene ketolase and β-carotene hydroxylase on the membrane, via their fusion by the glycerol uptake facilitator protein, resulted in a 2.2-fold improvement in the astaxanthin yield [42]. The protein scaffold strategy using heterotrimeric DNA sliding clamp PCNA (Proliferating Cell Nuclear Antigen) of *Sulfolobus solfataricus* P2 was also utilized to improve the activity of cytochrome P450 enzyme on the production of caffeic acid [43].

**Figure 2:**
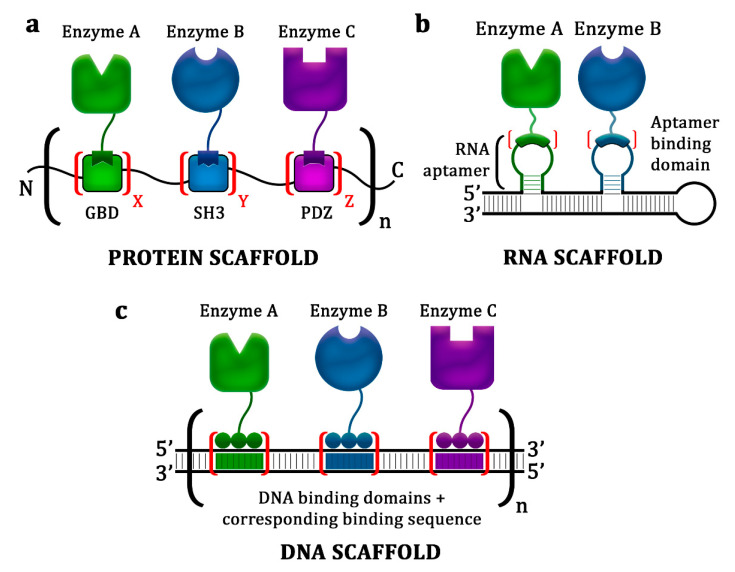
General approaches using the synthetic scaffolding strategy. (**a**) Protein scaffold system, where metazoan peptide motifs (i.e., GTPase-binding domain (GBD), Src homology 3 (SH3), and PSD95/Discs Large/ZO-1 (PDZ)) were exploited as scaffolds to anchor pathway enzymes that had been fused with peptide ligands associated with those domains. (**b**) RNA scaffold system, where synthetic noncoding RNAs containing aptamer structures were used as scaffolds to localize target enzymes that had been fused with appropriate aptamer-binding domains. (**c**) DNA scaffold system, where plasmid DNAs were used as scaffolds to dock target enzyme–DNA-binding protein (zinc finger protein of transcription activator-like effectors) fusions.

Perhaps one of the most successful protein-scaffolding approaches was the manipulation of the interaction between metazoan peptide motifs and their cognate adaptor domains. In this strategy, protein domains such as Src homology 2, Src homology 3, GTPase-binding domain, and PSD95/Discs Large/ZO-1 (PDZ), were used as scaffolds to anchor target enzymes that had been fused with peptide ligands associated with those domains (Figure 2a) [44]. This scaffolding strategy resulted in an increase in product yield ranging from 2.5- to 77-fold [37,38,45].

The highest enhancement of metabolic pathway efficiency, achieved using a protein scaffold system, was the 77-fold increase in mevalonate yield reported by Dueber et al. [25]. Those authors varied the number of protein domains in the scaffold to obtain the best ratio of anchored enzymes. Similar protein scaffold structures have been used for enhancing the d-glucaric acid, resveratrol, hydrogen, and butyrate production pathways [33,34,36], although only a maximum 5-fold enhancement in productivity was observed, which proved that variations among optimal scaffold architectures for different synthetic pathways can occur [46]. Thus, clarification of the exact mechanism behind the protein scaffold system is critical to realizing its full potential for efficiently enhancing metabolic pathways. Moreover, knowledge about the protein scaffold structure is also important for addressing the stability of the systems. As their complexity and size increase, full-length and functional protein scaffolds become difficult to express and prone to undesirable cross interactions, such as misfolding, aggregation, and degradation [29].

## 3. Nucleic Acid Scaffolds

Compared with proteins, nucleic acids are more predictable in structure and easier to manipulate [8]. Furthermore, the ready availability of bioinformatic tools (e.g., the Mfold and RNAfold web servers) for designing synthetic nucleic acid structures, along with the wide range of nucleic acid-binding domains and aptamers, allows the construction of scaffold systems that can arrange bound protein in a specific manner [47,48,49]. RNA and DNA scaffolds can be manipulated easily for a higher degree of flexibility, simply by changing the distance between the protein-binding sites and/or the adding polymerization domains [23]. Similar to the protein scaffold systems, nucleic acid scaffolds have already been applied on various biosynthetic pathways (Table 2).

### 3.1. RNA Scaffolds

**Figure 3:**
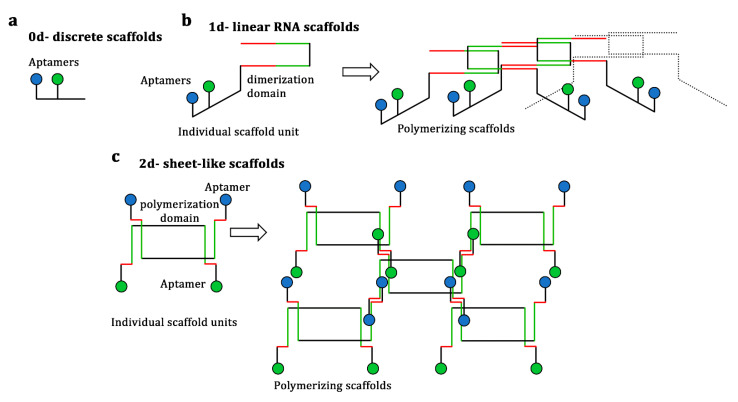
Multi-dimensionality of RNA scaffold systems. (**a**) In the case of a discrete RNA scaffold (zero dimension (0D)), a single RNA strand folds into a scaffold unit, generally presenting two aptamers for target pathway enzyme docking. (**b**) In the case of a one-dimensional (1D) RNA scaffold, each individual scaffold unit presents two aptamers and dimerization domains (red and green strands), which allow the individual units to form linear chains of aptamer sites through complementary base pairing. (**c**) In the case of a two-dimensional (2D) RNA scaffold, two different RNA strands, each presenting two aptamers, come together to form individual “tile” units. The polymerization domains (red strands) enable the polymerization of those units, resulting in the formation of 2D sheet-like structures through complementary base pairing [51].

RNA scaffold systems for the binding of target enzymes are based mainly on aptamers, which are short synthetic single-stranded oligonucleotides that bind specifically to various molecular targets (Figure 2b) [55]. The binding itself is facilitated by peptide or protein domains specific to the corresponding aptamer, which are fused to the enzyme of interest [56]. The application of individual RNA scaffold units (zero dimension) for docking hydrogenase and ferredoxin resulted in a 4-fold improvement in hydrogen production compared with that obtained by the non-scaffolded enzymes (Figure 3) [50]. The addition of dimerization and polymerization domains on the individual scaffold units enables the formation of linear chains (one dimension) and sheet-like structures (two dimensions), which improve the scaffold system efficacy (Figure 3b,c). Such one-dimensional and two-dimensional scaffolds have enhanced hydrogen yields by 8- and 48-fold, respectively [50]. These remarkable enhancements in the product yield are due to the increased number and architectural complexity of the scaffolds, as further evidenced by the 88% improvement in succinate biosynthesis through the anchoring of four enzymes by an RNA scaffold with a two-dimensional architecture [51].

To ensure metabolic efficacy, it is critical that the orientation of the enzyme active site is controlled in order to channel intermediates directly toward the next enzyme in the cascade [8]. The orientation can be adjusted by changing the aptamer stem-loop length (i.e., the number of base pairs), with one base-pair change being equivalent to a 30° change in the enzyme orientation [8,51]. The maintenance of a 120° angle between acyl-ACP reductase (AAR) and aldehyde deformylating oxygenase (ADO) resulted in the highest pentadecane production yield, with a 2.4-fold improvement over the yield by the natural system [51].

The application of RNA scaffold systems are not only limited to co-localizing metabolic enzymes. A system, termed as chaperone-recruiting mRNA scaffold (CRAS), is utilized to direct protein folding and translation machinery for preventing the aggregation and further misfolding of newly synthesized proteins. In CRAS system, 3′ untranslated region of mRNA of target protein is modified as a scaffold to anchor the bacterial chaperone DnaJ [52]. Using the system, about 90% of expressed *E. coli* UDP-glucose dehydrogenase (UGD), 80% of expressed Anti-Ras single-chain variable fragment (anti Ras-ScFv), and 50% of expressed *S. cerevisiae* alcohol dehydrogenase 1 (Adh1p) were soluble [52].

However, although RNA scaffolds are easy to design, can be built to have different geometrical compartments, and increase the product yield of various metabolic pathways, the RNA itself is still prone to misfolding and degradation, especially in larger and complex scaffold systems [15,47]. One research team addressed these problems by minimizing the single-stranded regions in the design, locking both ends of the RNA scaffold with hairpins, and using RNase E-knockout strains for expression (e.g., *E. coli* BL21 DE3 Star; Invitrogen) [57].

### 3.2. DNA Scaffolds

Plasmid DNA-based scaffolds offer a more stable platform for docking metabolic enzymes. Because the in vivo stability of plasmid DNA is generally sequence-independent, numerous architectures with virtually any sequence and length can be constructed without decreasing the availability of the scaffold (Figure 2c) [12,29]. Furthermore, recent advancements in genome editing have provided various molecular tools for executing highly effective and sequence-specific DNA targeting, such as zinc finger proteins (ZFPs), transcription activator-like effector (TALE) proteins, and clustered regularly interspaced short palindromic repeats, and its associated protein (CRISPR-Cas) [58,59]. Most of the DNA scaffold systems that have been reported utilized ZFPs for facilitating the binding of target enzymes because these proteins have high binding specificity with nanomolar dissociation constants and an easily constructed modular design. For instance, four-fingered ZFPs that recognize orthogonal 12-bp DNA sequences with no specific binding site in the host genome were selected from more than 2 × 10^6^ possible combinations [9,47]. Using this strategy, DNA scaffold systems have been successfully utilized to improve the yields of mevalonate, 1,2-propanediol, resveratrol, l-threonine, and *n*-alkane in *E. coli* and that of N-acetylglucosamine in Bacillus subtilis [9,25,30,47].

Optimal utilization of the DNA scaffold system was achieved through fine tuning of the spacing and stoichiometry of the enzymes on the scaffold. Because the double-helical structure of DNA turns 360° every ~10 bp, the addition of a 1-bp spacer between enzymes on the scaffold adds a 36° turn in the axial view [9]. Consequently, when the distance between enzymes on the scaffold is set by a multiple of 10 bp, the enzymes would be in the same plane, which supports efficient transfer of the intermediates. For mevalonate, 1, 2-propanediol, and resveratrol production, all scaffolds with 4-bp spacers between the zinc finger-binding sites were less effective than their 2- and 8-bp counterparts [22] owing to the enzymes on the scaffold being in opposite positions to each other when 4-bp spacers were applied. Spacing also determines the proximity of the enzymes to each other, which is important given that metabolic enzymes have to be close enough to allow effective substrate channeling for enhanced product synthesis [12]. In the case of l-threonine biosynthesis, having all enzymes in the same orientation on a scaffold with 8-bp spacers (20-bp distance between each enzyme–ZFP fusion) resulted in the highest production efficiency compared with that on scaffolds with 18- and 28-bp spacers [9].

**Figure 4:**
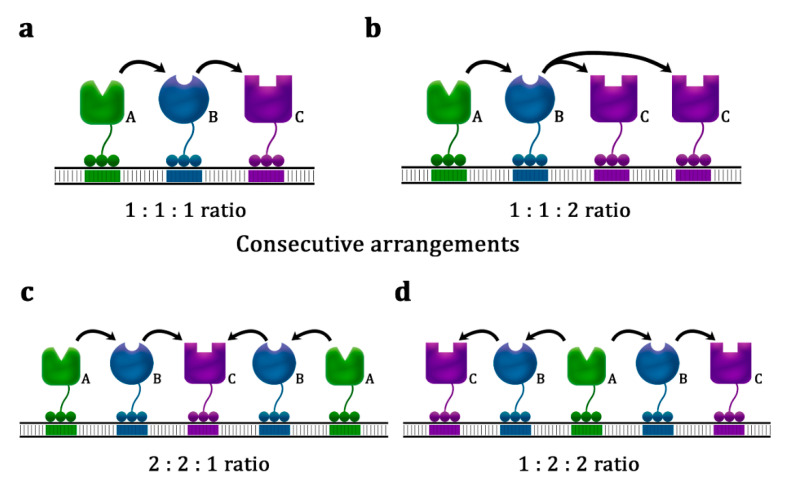
Examples of various DNA scaffold architectures. (**a**,**b**) Consecutive arrangement, where enzymes are arranged linearly in a [1:1:1] (**a**) and [1:1:2] ratio (**b**). (**c**,**d**) Bidirectional arrangement, where the third enzyme (C) is flanked on both sides by the second (B) and then the first (A) enzymes in a [2:2:1] ratio (**c**) or where the first enzyme (A) is flanked on both sides by the second (B) and then the third (C) enzymes in a [1:2:2] ratio (**d**).

Optimization of the enzyme stoichiometry on a scaffold involves control of the ratio between the enzyme-binding sites on a single scaffold unit and the number of scaffold unit repeats (e.g., described as (E1a:E2b:E3c)n for a three-enzyme scaffold, where a, b, and c represent the number of each enzyme within a single scaffold unit, and n is the number of times the scaffold unit is repeated on the plasmid) [12,47]. There are benefits to adjusting the enzyme arrangement on the scaffold (Figure 4a,b), especially for biosynthetic pathways that have enzymes with different kinetics. The highest n-alkane productivity (8.8 times higher than the control strain) was achieved when the AAR- and ADO-binding site ratio on a DNA scaffold was adjusted to [1:3] [25]. By contrast, for l-threonine production, the [1:1:2] arrangement of homoserine dehydrogenase, homoserine kinase, and threonine synthase yielded the highest productivity, followed closely by the [1:1:3] and [1:1:2] arrangements, and was 3-fold higher than that of [1:1:2] [9]. A case-by-case approach was also adopted for determining the optimal scaffold unit repetitions [47]. In general, the optimal stoichiometry of enzymes in DNA scaffolds varies depending on the pathway.

The TALE-based DNA scaffold system was developed as an alternative to the ZFP-based system [53]. In this system, enzymes were fused with artificially designed and constructed TALE domains and localized to their cognate sequence on the scaffold. Unlike the predefined sequences recognized by zinc finger domains, the 16-bp sequences cognate to TALE domains could be freely designed, emphasizing the flexibility of the TALE-based DNA scaffold system [60]. With the optimal enzyme ratio and arrangement, this system successfully improved mevalonate production by 3.7-fold [24]. Furthermore, an up to 9.6-fold yield increase was observed when this system was used for spatial organization of the enzymes involved in indole-3-acetic acid biosynthesis [53].

In general, DNA scaffold systems are more stable and easier to design than protein and RNA scaffold systems [8,29]. Simple modification of the plasmid DNA sequence allows rapid and easy optimization of the enzyme stoichiometry, spacing, orientation, and order. This system does have drawbacks, however, such as the propensity of plasmid DNA to become supercoiled, which might prevent enzymes from binding. Additionally, the presence of repetitive sequences of enzyme-binding sites in the plasmid DNA might result in recombination of the plasmid [29,47]. Furthermore, the DNA scaffold system is limited in terms of its structural flexibility; that is, since the anchored enzymes are arranged linearly on the scaffold, only intermediates passing through that particular “line” would come across a downstream enzyme, which limits the output of the metabolic reaction [15]. Nonetheless, the emergence of DNA origami technology has enabled the formation of higher-order DNA structures, similar to those of the RNA scaffold system, which might increase the efficacy of the DNA scaffold system [61,62].

## 4. Conclusions

With the advent of metabolic engineering and synthetic biology, it has become possible to introduce heterologous and/or synthetic enzymes into host organisms to “force” them to produce heterologous chemicals that they would otherwise not naturally produce in high quantities, if at all. However, enzymes with different reaction kinetics require optimization for balancing the metabolic flow and for reducing any negative impact on the host organism. Attempts to address these issues using traditional approaches, such as the rational design of expression systems and control of the enzyme expression level, have been met with limited success. By contrast, the complementary approach of controlling the spatial organization of pathway enzymes via synthetic scaffolds has proven more effective in solving the problems related to inefficient cascade reactions, low biosynthetic yields, and reduced host viability.

Although protein and RNA scaffold systems are able to improve the efficiency of metabolic pathways by up to 77- and 48-fold, respectively, such outputs require highly complex scaffold structures, which are prone to misfolding and degradation. By contrast, DNA scaffold systems offer higher structural stability, but their limited structural flexibility results in generally lower enhancements of metabolic pathways.

Current efforts toward increasing the efficiency of metabolic pathways via synthetic scaffold systems have mostly involved laborious trial-and-error processes with multiple factors to consider, such as the number of scaffolds to use and the optimal design of the arrangement, orientation, and spacing of the enzymes. Further investigation is needed on the exact mechanisms underlying the beneficial effects of scaffold systems on metabolic pathways. Specifically, in-depth studies on the structural biology of substrate channeling would provide valuable information for aiding the future design of simple, accurate, and tunable synthetic scaffold systems.

## Figures and Tables

**Table 1:** Examples of protein scaffold systems.

Product	Scaffolded Enzyme	Domains	Host	Maximum Fold Increase ^1^	Reference
**Domains and Ligands from Metazoan Cells**					
Mevalonate	Acetoacetyl-CoA thiolase, hydroxymethylglutaryl-CoA synthase, hydroxymethylglutaryl-CoA reductase	GBD, SH3, PDZ	*Escherichia coli*	77	[32]
d-Glucaric acid	Myo-inositol-1-phosphate synthase, myo-inositol oxygenase	SH3, PDZ	*E. coli*	3	[32]
d-Glucaric acid	Myo-inositol-1-phosphate synthase, myo-inositol oxygenase, uronate dehydrogenase	GBD, SH3, PDZ	*E. coli*	5	[33]
Hydrogen (H_2_)	[Fe-Fe]-hydrogenase, ferredoxin	SH3, PDZ	*E. coli*	3	[34]
Resveratrol	4-Coumarate- CoA ligase, stilbene synthase	SH3, PDZ	*Saccharomyces cerevisiae*	5	[35]
Butyrate	3-Hydroxybutyryl-CoA dehydrogenase, 3-hydroxybutyryl-CoA dehydratase, *trans*-enoyl-coenzyme A reductase, acyl-CoA thioesterase II	GBD, SH3, PDZ	*E. coli*	3	[36]
Gamma-aminobutyric acid (GABA)	Glutamate decarboxylase, glutamate/GABA antiporter	SH3	*E. coli*	2.5	[37]
Catechin	Flavanone 3-hydroxylase, dihydroflavonol 4-reductase, leucoanthocyanidin reductase	GBD, SH3, PDZ	*E. coli*	1.3	[38]
Itaconic acid	Citrate synthase, acotinase, cis-acotinic acid decarboxylase	GBD, SH3, PDZ	*E. coli*	3.8	[39]
Malic acid	Phosphoenolpyruvate carboxylase, malate dehydrogenase	SH3	*E. coli*	3.6	[40]
Indigoidine	Glutamine synthetase, indigoidine synthase (indC), helper protein of IndC (IndB)	GBD, SH3, PDZ	*E. coli*	2.1	
**Cohesin and dockerin domains**					
NADH	Alcohol dehydrogenase, formaldehyde dehydrogenase, formate dehydrogenase	Cohesin–dockerin pairs from *Clostridium cellulolyticum*, *Clostridium thermocellum*, and *Ruminococcus flavefaciens*	*S. cerevisiae*	5	[30]
2,3-Butanediol	Acetolactate synthase, acetolactate decarboxylase, 2,3-butanediol dehydrogenase	Cohesin–dockerin pair from *C. thermocellum*	*S. cerevisiae*	1.4	[31]
**Other domains**					
1-Butanol	3-Hydroxybutyryl-CoA dehydrogenase, crotonase, butyryl-CoA dehydrogenase, butylaldehyde/butanol dehydrogenase	Leucine zipper domains	*E. coli*	1.5	[41]
Astaxanthin	β-Carotene ketolase, β-carotene hydroxylase	Glycerol uptake facilitator protein	*E. coli*	2.2	[42]
Caffeic acid	ferredoxin, ferredoxin reductase, cytochrome P450 enzyme	PCNA of *Sulfolobus solfataricus* P2	*E. coli*	8	[43]

^1^ Compared with the natural system without the scaffold. GBD, GTPase-binding domain; SH3, Src homology 3; PDZ, PSD95/Discs Large/ZO-1; PCNA, Proliferating Cell Nuclear Antigen.

**Table 2:** Examples of nucleic acid scaffold systems.

Product	Scaffolded Enzyme/Protein	Nucleic Acid-Binding Protein	Host	Maximum Fold Increase ^1^/Other Result	Reference
**RNA Scaffold Systems**					
Hydrogen (H_2_)	[Fe-Fe]-hydrogenase, ferredoxin	PP7, MS2	*Escherichia coli*	48	[50]
Pentadecane	Acyl-ACP reductase, aldehyde deformylating oxygenase	BIV-Tat, PP7	*E. coli*	1.8	[51]
Succinate	Carbonic anhydrase, pyruvate carboxylase, malate dehydrogenase, NAD-formate dehydrogenase	RevR11Q, PP7, BIV-Tat, Lambda N	*E. coli*	2.6	[51]
*E. coli* UDP-glucose dehydrogenase (UGD), Anti-Ras single chain variable fragment (anti-Ras ScFv)	DnaJ chaperone	KH3	*E. coli*	90% of expressed UGD and 80% of expressed anti-Ras ScFv were soluble	[52]
**DNA scaffold systems**					
*trans*- Resveratrol	4-Coumarate–CoA ligase, stilbene synthase	Zif268, PBSII (3 fingers ZFP)	*E. coli*	3	[47]
1,2- Propanediol	Methylglyoxal synthase, 2,5-diketo-d-gluconic acid reductase, glycerol dehydrogenase	ZFa, ZFb, ZFc (3 fingers ZFP)	*E. coli*	4	[47]
Mevalonate	Acetoacetyl-CoA thiolase, hydroxymethylglutaryl- CoA synthase, hydroxymethylglutaryl-CoA reductase	ZFa, ZFb, ZFc (3 fingers ZFP)	*E. coli*	3	[47]
l-Threonine	Homoserine dehydrogenase, homoserine kinase, threonine synthase	ADB1, ADB2, ADB3 (4 fingers ZFP)	*E. coli*	Reduced production time by more than 50%	[9]
*n*-Alkane	Acyl-ACP reductase, aldehyde deformylating oxygenase	ADB2, ADB4 (4 fingers ZFP)	*E. coli*	8.8	[25]
Indole-3-acetic acid	Tryptophan-2-mono-oxygenase, indole-3-acetamide hydrolase	TALE1, TALE2	*E. coli*	9.6	[53]
Mevalonate	Acetoacetyl-CoA thiolase, hydroxymethylglutaryl- CoA synthase, hydroxymethylglutaryl-CoA reductase	TALE1, TALE2, TALE3	*E. coli*	3.7	[54]

^1^ Compared with the natural system without the scaffold. PP7, *Pseudomonas aeruginosa* phage PP7; MS2, *E. coli* phage MS2; BIV-Tat, bovine immunodeficiency virus Tat protein; KH3, human Nova-1 KH3 domain; Zif268, early growth response protein 1; ZF, zinc finger; ZFP, zinc finger protein; ADB, artificial DNA-binding domain; TALE, transcription activator-like effector.
